# The Covariance between Air Pollution Annoyance and Noise Annoyance, and Its Relationship with Health-Related Quality of Life

**DOI:** 10.3390/ijerph13080792

**Published:** 2016-08-06

**Authors:** Daniel Shepherd, Kim Dirks, David Welch, David McBride, Jason Landon

**Affiliations:** 1 School of Public Health, Auckland University of Technology, Private Bag 92006, Auckland 1142, New Zealand; jason.landon@aut.ac.nz; 2 School of Population Health, University of Auckland, Private Bag 92019, Auckland 1142, New Zealand; k.dirks@auckland.ac.nz (K.D.); d.welch@auckland.ac.nz (D.W.); 3 Department of Preventive & Social Medicine, University of Otago, P.O. Box 56, Dunedin 9054, New Zealand; david.mcbride@otago.ac.nz

**Keywords:** environmental noise, air pollution, health related quality of life (HRQOL), noise annoyance, covariance, traffic

## Abstract

Air pollution originating from road traffic is a known risk factor of respiratory and cardiovascular disease (both in terms of chronic and acute effects). While adverse effects on cardiovascular health have also been linked with noise (after controlling for air pollution), noise exposure has been commonly linked to sleep impairment and negative emotional reactions. Health is multi-faceted, both conceptually and operationally; Health-Related Quality of Life (HRQOL) is one of many measures capable of probing health. In this study, we examine pre-collected data from postal surveys probing HRQOL obtained from a variety of urban, suburban, and rural contexts across the North Island of New Zealand. Analyses focus on the covariance between air pollution annoyance and noise annoyances, and their independent and combined effects on HRQOL. Results indicate that the highest ratings of air pollution annoyance and noise annoyances were for residents living close to the motorway, while the lowest were for rural residents. Most of the city samples indicated no significant difference between air pollution- and noise-annoyance ratings, and of all of the correlations between air pollution- and noise-annoyance, the highest were found in the city samples. These findings suggest that annoyance is driven by exposure to environmental factors and not personality characteristics. Analysis of HRQOL indicated that air pollution annoyance predicts greater variability in the physical HRQOL domain while noise annoyance predicts greater variability in the psychological, social and environmental domains. The lack of an interaction effect between air pollution annoyance and noise annoyance suggests that air pollution and noise impact on health independently. These results echo those obtained from objective measures of health and suggest that mitigation of traffic effects should address both air and noise pollution.

## 1. Introduction

Population growth, an increasing demand for consumer goods, and sustained urbanization have led to concerns over the lived environments in many of the world’s cities. Environmental quality is an important determinant of health [1] and, in recent times, attention has been drawn to the influence of traffic-related air and noise pollution on health outcomes, especially with respect to at-risk groups, both in relation to long-term exposure [2] as well as acute effects from brief exposures [3]. Though sharing a common source, the traditional approach to studying the health risks associated with exposure to traffic air pollution and traffic noise has been to study them in isolation [4]. This approach may possibly be because the urban form makes it difficult to model exposures accurately and thus additional modelling requires greater investment of resources, or because when it comes to health effects, there is a belief that they have unique pathways. Increasingly, however, studies are broadening to include coexistent pollutants, for example, both air pollution and noise in relation to cholesterol levels [5], and in relation to myocardial infarction [2]. As appears to be common in other studies as well, both Sorensen et al. [5] and Tonne et al. [2] found significant effects for air pollution and more modest associations for noise after controlling for air pollution [2,5]. Limitations identified in such studies tend to indicate a difficulty in separating air pollution from noise because of the high degree of correlation between the two when in close proximity to the source [6,7]. Indeed, recent attempts have taken advantage of their covariation, as well as the low cost associated with collecting noise measurements compared with air pollution, to demonstrate the potential for noise exposure to be used as a surrogate for air pollution exposure [8]. There may be merit to this approach given the reported associations between noise annoyance and perceptions of air quality (e.g., [9]).

### 1.1. The Health Effect of Noise and Air Pollution

Air pollutants can be detected either visually, such as witnessing smoke emanating from a vehicle’s exhaust, or by smell such as when odorants stimulate olfactory receptors. The evidence linking air pollution to adverse impacts on human health is now extensive [10,11]. The WHO [12] estimates that globally in 2012 alone, 3.7 million deaths were attributable to ambient air pollution (i.e., PM_10_ and PM_2.5_). A study undertaken in The Netherlands indicated that the Disability Adjusted Life Years (DALYs) attributable to particulate air pollution contributed 60% of the burden of disease associated with environmental factors [13]. However, the findings of research into the effects of air pollution on mental health are less conclusive. For example, analyses of large European datasets indicates that exposure to air pollution is not associated with depressed mood [14], while other studies have found links between air pollution and perceived stress [15].

Many air pollution health studies have focused specifically on urban areas, and vehicle-generated pollution in particular, as road vehicles are one of the major sources of air pollution across much of the world. Elemental carbon, NOx and ultrafine particles are considered to be pollutants most strongly associated with road traffic emissions [16]. In Auckland (New Zealand), it has been estimated that 71% of summer and 21% of winter concentrations of fine particulate matter is attributable to motor vehicles [17]. Moreover, poor town planning decisions in Auckland (and in New Zealand in general) over many decades has meant that many people live in very close proximity to busy roads and motorways (or within “road corridors”), and so are vulnerable to the adverse effects of road traffic, including noise and air pollution as well as experiencing a potential for degradation in their quality of life and the perceived amenity of their surroundings. As such, New Zealand is highly suitable for studies investigating the impact of roads on the health of its residents.

Equally, exposure to high-intensity noise can lead to hearing loss, while noise below the levels effecting hearing loss can interfere with an organism’s revitalization processes (e.g., sleep or rest), and cognitive processes requiring directed attention in order to realize adaptive behavior. Furthermore, there is weak evidence suggesting a link between noise and both depression and anxiety disorders [18]. Others report that residential noise exposure is associated with both self-assessed health and mental health status [19]. Substantial exposure to road traffic noise [20,21] and aviation noise [22] has also been linked to cardiovascular disease, hypertension, and ischemic heart disease. Elucidating the relationship between noise and health has been somewhat thwarted by measurement issues pertaining to noise at both the psychological (e.g., [23]) and physical (e.g., [24]) levels of description. At the psychological level, individual vulnerabilities to noise (i.e., noise sensitivity) confound a simple one-to-one mapping of the noise-to-human response; and noise sensitivity has often been found to be a better predictor of the health impacts of noise than the noise levels themselves [25]. Consequently, simple bivariate relationships between noise level and human response (e.g., annoyance) are no longer considered valid approaches to the protection of the health of the public [24,26]. Arguably, degrees of annoyance to noise is not readily represented by current “best-practice” sound metrics [27]; instead, they can only be described by those exposed to the noise. Thus, Dratva et al. [28] suggest that measures of noise annoyance may be superior to noise level when mitigating the harmful impacts of noise. Considering air pollution, Jacquemin et al. [29] similarly argued that annoyance measures could be used as complementary tools for health surveillance.

### 1.2. Combined Effects of Air and Noise Pollution on Health

With the exception of electrified trains, transport-related air pollution is usually accompanied by some degree of noise emanating from the same source. This presents a challenge when attempting to estimate the negative contribution of each source to population health, and determining whether they degrade health independently (i.e., unique effects) or in some manner interact (i.e., combined effects). Previous studies have demonstrated strong combined impact of noise and air pollution on annoyance ratings (e.g., [30]). Stansfeld [18], commenting on the difficulties of disentangling the effects of the two exposures on account of their substantial covariance, suggests that independent effects may be more readily gauged by seeking out geographical regions with weaker correlations. For example, rural areas are characterized by lower road-traffic volumes, and air pollution is more efficiently removed from the environment due to meteorological conditions which may not impact noise. Furthermore, rural areas have low ambient noise levels which can increase the saliency of intermittent noise [31] due to an increased potency of sound events to direct attention [32]. Compounding the issue, noise may travel further in these rural regions due to a lack of intervening infrastructure [33].

A further consideration rests on the mechanisms by which each exposure may affect health as there are key differences between the two exposures. The two types of exposure utilize different biological pathways to affect functioning and compromise health. In a narrative review, Stansfeld [18] concluded that noise increases morbidity and mortality independently of air pollution exposure, though air pollution constituted a greater burden of disease whilst noise exposure had a greater impact on quality of life. However, anatomical and physiological findings indicate that the two separate pathways may share a common destination as both are associated with an increase in sympathetic activity [18], and ultimately, both noise and air pollution can be considered environmental stressors [34]. Stress responses, in turn, are known to be better predicted by subjective assessments of the stressor, by resilience levels, and by an individual’s coping style. However, a recent paper by von Lindern, Hartig, and Lercher [35] emphasizes the negative impact of traffic noise and air pollution upon the restorative potency of homes, which may exceed the “stressor” effects of such exposures. Furthermore, it has been found that self-report health symptoms are not well explained by the level of exposure to odorous air pollutants, rather the relationship is mediated by perceptions of exposure and health risk [36]. Similar findings are found in relation to noise exposure and health-related quality of life [37], indicating that the underlying mechanisms relating pollutant exposure and HRQOL maybe common to both noise and air pollution. This convergence to common physiological or psychosocial mechanisms introduces further complexity when trying to tease apart the unique effects of air pollution and noise upon health and health-related quality of life.

### 1.3. Environmental Stressors and Health Effects

While exposure to polluted air is accepted as an adverse health effect [38], the debate around whether environmental noise degrades health and, if so, whether this effect is direct or indirect, is still not agreed upon by all. In the air pollution context, Sherwin [39] defines an adverse health effect as any agent implicated with the “causation, promotion, facilitation and/or exacerbation of a structural and/or functional abnormality, with the implication that the abnormality produced has the potential of lowering the quality of life, contributing to a disabling illness, or leading to a premature death” (p. 177). Evidently, this definition embraces both direct and indirect health effects, and, holding this criterion against the existing evidence (e.g., [40,41]), it can be argued that noise constitutes an adverse health effect. Furthermore, the WHO explicitly categorizes cognition and sleep as direct indicators of health [42]. Agents directly modifying these two processes must therefore be considered direct health effects. Noise can directly impact both cognition and sleep, and therefore noise, like air pollution, must be considered to cause a direct health effect above and beyond hearing loss.

**Figure 1:**
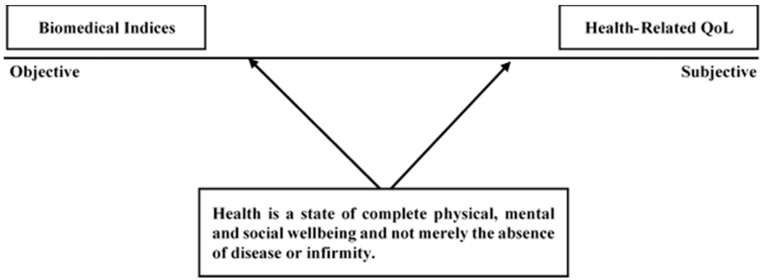
Approaches to health assessments. Subjective evaluations (i.e., HRQOL) capture the wellbeing aspects of health, while objective biomedical measures index the disease and infirmity aspect.

Accepting that both air pollution and noise can impact negatively upon health, one question is how to measure the independent (i.e., unique) and interactive (i.e., combined) effects of the two. This becomes a question of statistical method, but a more important question is how to measure health itself. Health is defined most authoritatively by the WHO (1948) as “…a state of complete physical, mental and social well-being and not merely the absence of disease or infirmity” [43]. However, this is a conceptualization of health, not an operationalisation, and does not explicitly instruct on measurement approaches. Figure 1 displays this conceptualization of health alongside approaches to its operationalisation, which can be modelled as a continuum anchored by two polar opposites: objective versus subjective health assessment approaches. Note that as a continuum, these two approaches should not be considered mutually exclusive, and there is some overlap across adjacent categories. Subjective measurements are those that rely on “first-person” evaluations of personal health, including self-referential Health-Related Quality of Life (HRQOL), measures assessing an individual’s physical, social, and psychological capacities. The WHO define QOL as:

*An individual's perception of his/her position in life in the context of the culture and value systems in which he/she lives, and in relation to his/her goals, expectations, standards and concerns. It is a broad-ranging concept, incorporating in a complex way the person's physical health, psychological state, level of independence, social relationships, and their relationship to salient features of their environment*.

(WHOQOL Group, [44])

As such, HRQOL measures represent a broader and more idealistic approach to health (i.e., wellbeing, or the quality of health) beyond that of morbidity or mortality indices (i.e., quantity of health). Biomedical indices consist of those medical assessments that are typically mechanical, chemical, or electrical, such as spirometry, blood cortisol levels or heart function, respectively. These can be considered absolute measures, and international standards exist to guide the methods used to obtain them. For epidemiological data collected publically using surveys, as we report in this study, the HRQOL approach has specific advantages in terms of ease of data collection and low cost, as well as using a “first-person” approach to health assessment. Furthermore, Vienneau et al. [45] note that, while air pollution is related to mortality measures and noise pollution to morbidity and degraded quality of life, in terms of total external costs, the burdens are equivalent.

### 1.4. Study Objectives

The review of Stansfeld [18] focused on the relative contribution of noise and air pollution exposure to objective health outcomes such as hypertension and atherosclerosis. One paper [46] bemoans a lack of studies examining the concurrent effects of air and noise pollution on aspects of mental health. In this paper, we focus on the relative contribution of noise and air pollution annoyance on subjective health outcomes in the form of HRQOL, which includes measures of psychological wellbeing. The study has four primary aims, focusing on annoyance to air pollution and noise generated by road traffic in both urban and rural areas. The first is to measure the degree of annoyance to air pollution and noise across urban and rural environments. Here, we predict that the urban samples will have comparatively equal exposures to air and noise pollution, and the combined effect [30] will lead to equivalent self-reported levels of annoyance. On the other hand, for rural areas where exposures are not expected to be equivalent, noise should be judged as more annoying. The second aim is to measure correlations between air pollution annoyance and noise annoyance. Here, we predict that correlations will be greater for urban areas than for rural areas. Thirdly, we aim to examine the associations between air pollution annoyance and noise annoyance and the four domains of HRQOL. Lastly, we apply statistical methods to estimate the independent effects of each upon HRQOL. Based on Stansfeld [18], we hypothesise that noise annoyance has a stronger impact on HRQOL than air pollution annoyance.

## 2. Materials and Methods

### 2.1. Participants

The data for this study were collected in New Zealand’s two largest cities: Auckland (in 2010 and 2014) and Wellington (in 2010 and 2012). The Auckland data consisted of two datasets. The first set compromised the “Motorway” (*n* = 373) and the “Non-Motorway” (*n* = 253) samples. The Motorway sample consisted of residents living within 50 m of Auckland’s motorway system, with noise levels estimated to be approximately 76 dB(A) LDN [47]. The Non-Motorway reference sample contains data from residents from two areas within the Auckland region, located at least two kilometres away from any significant source of environmental noise (e.g., industry or roads), and with noise levels estimated to be around 55 dB(A) LDN. The second Auckland dataset consisted of data from a central suburb, denoted “CBD-Suburb” (*n* = 173) which contained a number of main thoroughfares giving passage to and from the central city. In the CBD-Suburb area the noise levels were estimated to be approximately 60 dB(A) LDN. The Motorway and Non-Motorway samples were socioeconomically matched (middle to high deprivation) and were from suburban neighbourhoods, while the CBD-Suburban sample was a low deprivation area and occupies the urban/suburban boundary.

The Wellington data comprises the “Airport” sample (*n* = 87), the matched “Non-Airport” sample (*n* = 93), and the “Rural” sample (*n* = 171). The Airport sample consisted of responses from residents living within 250 m of the Wellington International Airport’s runway, with aviation noise levels estimated at 62 dB(A) LDN, and peak values legislated to stay below 75 dB(A) Lmax [48]. The Non-Airport reference sample consisted of residents living on the city’s urban/suburban border, and far from the airport’s main flight path. The Airport and Non-Airport samples were matched (low to middle deprivation), and both were neighbourhoods on the urban/suburban boundary. The Rural sample contained individuals living in rural areas immediately beyond the outskirts of Wellington, areas classified as low deprivation. Rudimentary demographic profiles for the six samples are presented in Table 1.

### 2.2. Questionnaire

Data were obtained from a larger survey entitled “Wellbeing and Neighborhood Survey” [47,49]. Items pertinent to the current analysis were the items probing annoyance to environmental factors, including traffic-related noise and air pollution, where “traffic” was unspecified but the context can be assumed to relate to road traffic. These items were presented by way of a five-point scale ranging from “not annoyed at all” to “extremely annoyed”. An additional “Other” category was included for both noise and air pollution sources, in which respondents were required to indicate other sources (e.g., aircraft or rail traffic). For these annoyance items, the respondents were given the following directive “Thinking about the last six months, how annoyed are you about the following?”. Health-related quality of life was estimated using the WHO’s short-form quality of life instrument, the WHOQOL-BREF [50]. This instrument presents two general items on self-rated health and quality of life, and 24 items representing four HRQOL domains: physical health (7 items), psychological wellbeing (6 items), social relationships (3 items), and environmental factors (8 items). Each WHOQOL-BREF item is rated using a five-point scale, with the higher-domain scores indicating more positive evaluations of HRQOL domain scores. It has been proposed that the WHOQOL-BREF is well suited for use in public health research [50], is well validated [51], and has been shown to have sound psychometric properties in noise research [47]. Participants were asked to rate their levels of sensitivity or resistance to noise using a three-point category scale (Not noise sensitive/Moderately noise sensitive/Very noise sensitive). The final section of the survey sought personal information including gender, age, years of residence and whether they were currently diagnosed with an illness.

Each eligible household had two surveys deposited in their letterboxes, along with pre-paid, return-addressed envelopes. A cover sheet explained who was conducting the survey and for what purpose, and invited potential participants to take part in research investigating their place of living and wellbeing. The title of the surveys, “Wellbeing and Neighborhood Survey” was designed to disguise the true intent of the study in order to minimize self-selection biases. Respondents completed the surveys independently in their own time and in their own homes, and no incentives were offered for their return. These studies were approved by the Auckland University of Technology Ethics Committee (08/256).

### 2.3. Statistical Analysis

Data analyses were carried out using SPSS Version 22 (IBM Corp., Armonk, NY, USA). For each of the six areas, zero-order and partial correlation coefficients were obtained for the air pollution annoyance and noise annoyance measures. For the partial correlations, the covariates were age, education, gender, illness (current) and noise sensitivity. A hierarchical regression analysis was conducted to investigate the independent and moderating effects of air pollution annoyance and noise annoyance upon HRQOL. For this analysis, potential confounds (age, gender, education, noise sensitivity, current illness) were simultaneously entered into Step 1, followed by air pollution annoyance and noise annoyance in Step 2. In Step 3, the air pollution annoyance by noise annoyance interaction term was entered.

## 3. Results

### 3.1. Summary Statistics for Annoyance Data

**Figure 2:**
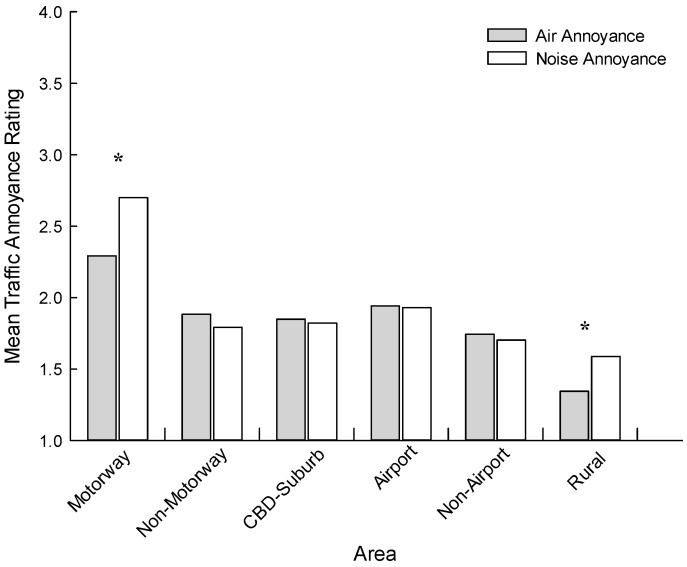
Mean air quality and noise annoyance ratings as a function of area. Asterisks indicate statistically significant differences (*p* < 0.001) between air quality and noise annoyance ratings within an area.

Figure 2 presents mean air quality and noise annoyance ratings across the six target areas. Only the Auckland Motorway (t (264) = −5.806, *p* < 0.001) and Wellington Rural (t (169) = −3.571, *p* < 0.001) samples returned statistically significant differences in their air quality and noise annoyance ratings. An additional factorial ANOVA was undertaken to test for mean air quality and noise annoyance differences across the six areas. In this model, area, education, current illness and noise sensitivity were included as between group factors, and age as a covariate. Considering air pollution annoyance, area (F (5, 988) = 5.275, *p* < 0.001), age (F (1, 988) = 20.018, *p* < 0.001) and noise sensitivity (F (2, 988) = 5.861, *p* < 0.001) were all significant. For area, Bonferroni post hoc tests revealed that the Rural sample was significantly lower than all other areas (*p* < 0.05), with no other pairwise comparisons reaching significance. Adapting the same model for noise annoyance, area (F (5, 987) = 13.949, *p* < 0.001), age (F (1, 987) = 10.673, *p* = 0.002) and noise sensitivity (F (2, 987) = 9.763, *p* < 0.001) were again significant. Post hoc tests indicated that the Motorway sample had a significantly higher mean annoyance than all other areas (*p* < 0.05), with no other pairwise comparisons reaching significance. 

### 3.2. Correlational Analysis

The relationship between air quality and noise annoyance was further examined using bivariate and partial correlational analyses. Across all six areas, a significant association was noted (*r* = 0.524, *p* < 0.001), even after controlling for age, education, gender, illness and noise sensitivity (*r* = 0.496, *p* < 0.001). Correlation coefficients for each of the six areas are presented in Table 2, where all values are significant (*p* < 0.001). Using Cohen’s [52] convention, the magnitudes of the coefficients are moderate to large, with Wellington’s Airport and Auckland’s Non-Motorway samples having the largest and smallest coefficients, respectively.

### 3.3. Summary Statistics for Health Related Quality of Life

**Figure 3:**
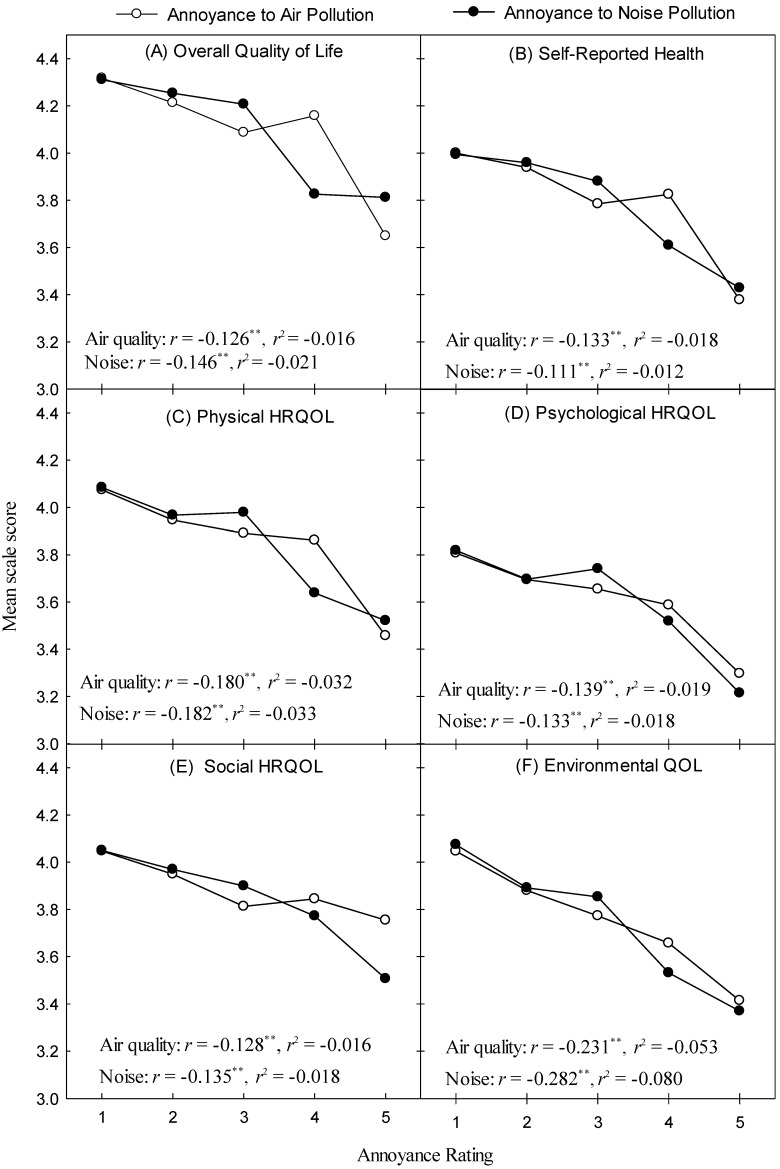
Plots of mean scale scores for outcome variables of interest versus air quality (open symbols) and noise annoyance (closed symbols). Each panel represents an aspect of HRQOL or self-reported health (Panel B). Asterisks indicate statistically significant differences (** *p* < 0.001).

Figure 3 plots mean overall QOL ratings (Figure 3A), self-reported health (Figure 3B), HRQOL (Figure 3C–E) and environmental QOL (Figure 3F) as a function of air quality and noise annoyance.

For all six plots, a general decrease in average scale scores occurs as annoyance increases. Partial correlation coefficients, controlling for Age, Education, Gender, Illness and Noise Sensitivity can be read from the interior of each plot. The magnitude of the correlation coefficients are small [52], and, in all cases, a quadratic model failed to provide a significant improvement over the results presented in Table 2. For air pollution annoyance, the weakest relationship was with overall QOL (Figure 3A) and the strongest with Environmental QOL, while for noise, it was self-reported health and Environmental QOL, respectively. Within any one outcome measure, corrected Fishers r-to-z transformations (two-tailed) revealed no significant differences in *r*-values between air quality and noise annoyance (*p* > 0.05), though Environmental QOL came close (*z* = 1.733, *p* = 0.083).

### 3.4. Multiple Linear Regression

A battery of hierarchical multiple linear regression analyses [53] were performed to determine the independent predictive value of air pollution annoyance, noise annoyance, and their interaction effect upon HRQOL and environmental QOL as represented by the four domains of the WHOQOL-BREF (see Table 3). In Step 1 of the regression analysis, Age, Education, Gender, Illness, and Noise Sensitivity were entered simultaneously to control for their effects. In Step 2, air pollution annoyance and noise annoyance were added to the model alongside the variables shown in Step 1, and in Step 3, their interaction term was added to all variables from Steps 1 and 2. Illness emerged as the dominant predictor for Physical and Psychological HRQOL, though air pollution annoyance and noise annoyance explained the second greatest portion of variance, respectively. While Education explained the most variability in Social HRQOL, noise annoyance was the dominant predictor of Environmental QOL. In all four analyses, air quality and noise annoyance explained significant amounts of variability in the dependent variable. The addition of the interaction effect failed to improve the model, indicating the absence of a moderating effect between air quality and noise annoyance. Follow-up simultaneous regression analyses excluding the interaction term demonstrated that inclusion of the interaction term made little-or-no difference to the regression coefficients presented in Table 3.

## 4. Discussion

A primary objective of this research was to document the degrees of air pollution annoyance and noise annoyance across areas differentiated by infrastructure, traffic volumes, and population density. As expected, the highest levels of annoyances were recorded in the Auckland Motorway sample, and the lowest in the Wellington Rural sample. Our prediction of equivalent air quality versus noise annoyance levels within samples obtained from city locales was largely upheld, though the Auckland Motorway example was an exception. In this sample, the mean noise annoyance ratings were significantly higher than the air pollution annoyance ratings. This prediction rested on the assumption that, while different sectors of the city may be exposed to differing levels of air and noise pollution, within each sector, the exposures would be roughly equivalent (i.e., the detection of air pollution would be proportionally related to noise exposure and vice versa). Speculatively, it may be that an interaction between the physical environment of the motorways and meteorological factors is effectively dispersing the air pollution, while no such attenuation of noise occurs. Such a situation would also occur in rural areas, albeit at lower exposure levels, and accordingly we predicted that for rural areas, noise annoyance would be greater than air pollution annoyance, as was the case for our data.

Further analysis of the association between air quality and noise annoyance ratings was undertaken using correlation techniques. As just discussed, with mean annoyance levels, it was predicted on the basis of exposure characteristics that correlations would be greater for the city areas (i.e., Motorway, CBD-Suburb, Airport, Non-Airport) than the suburban area (i.e., Non-Motorway) and the rural areas. This was supported, with the Auckland Motorway and Wellington airport samples having the highest correlation coefficients, and the Auckland Non-Motorway and Wellington Rural areas having the lowest. Taken as a whole, these differences in correlation coefficients indicate that exposure characteristics, and not personality factors (e.g., negative affect), may be driving annoyance responses. Additionally, our correlations between air quality and noise annoyance mirrored those reported for physical measures of air pollution and noise. Our range of correlations, from *r* = 0.292 to *r* = 0.633 are close to those reported in the literature, where Gan et al. [54] reported correlations between *r* = 0.18 and *r* = 0.48, and Tonne et al. [2] reports correlations as high as *r* = 0.54. This suggests that future research comparing subjective to objective measures may be of benefit.

Few studies have utilized HRQOL measures to assess the association between of air quality and noise and health, but those that have tend to report inverse relationships between the two (e.g., [55]). Qualitative analyses of our results (re: Figure 3) suggest a strong coupling between air pollution annoyance and noise annoyance on their effects on facets of quality of life. Disentangling the effects of the two sources of annoyance remains an ongoing challenge. Unsurprisingly, air quality and noise annoyance was associated most with Environmental QOL, though across all facets of QOL, a consistent negative trend was noted, that is, after controlling for relevant variables, an increase in annoyance was associated with a decrease in QOL. Thus, while it can be concluded that the effect of air quality and noise annoyance on QOL is modest, it is consistent with findings showing dose-response relationships between exposures and objective health measures such as hypertension (e.g., [56]).

Finally, our analyses of the independent effects of air pollution annoyance and noise annoyance on the four domains of the WHOQOL-BREF revealed that while air pollution annoyance predicted greater variability in physical HRQOL than noise annoyance ratings, the opposite was true for the psychological, social and environmental domains. For example, in relation to environmental QOL, noise annoyance had a greater impact than annoyance to air pollution. This may occur because while air pollution rapidly dissipates from its source, noise is more resilient and may travel further, including through intervening structures which may serve only to attenuate selective frequencies. This finding is consistent with Honold [57], who noted that perceived air pollution, but not noise pollution, predicted self-reported health. A further finding that warrants consideration is the lack of an interaction between air pollution annoyance and noise annoyance. This adds weight to the assertion that the impacts of air pollution and noise on self-reported health are largely independent [18,58]. This contrasts to findings indicating combined effects between noise and air pollution upon self-report annoyance [30].

A number of limitations inherent in this study need to be considered when judging both validity and generalizability. Firstly, the authors did not independently undertake noise level measurements in the selected areas, instead relying on third-party reports or models based on traffic intensity. However, as Birk et al. [59] report, significant associations between self-reported annoyance to road noise and GIS modelled road traffic noise exposure exist. Secondly, air pollution measurements were not undertaken at all, and the impact of traffic-related vibration was likewise not considered. Thirdly, the samples sizes from some of the areas was modest in light of the analytical choices, and greater external validity would have been obtained with increases in sample size. Lastly, wording of some survey items reflect a cultural bias that would limit their usefulness outside of New Zealand. For example, the term “traffic” can be considered to mean road traffic, and in everyday usage and in the New Zealand media terms such as “traffic jams”, “stuck in traffic” and “noisy traffic” will all without exception relate to road traffic. Thus why it may appear questionable that the data obtained from the airport sample relates to road traffic we are confident that we have captured the intended annoyance responses. Evidence can be marshalled from the open-ended responses required from participants selecting the “Other” category, which revealed that for the air quality item, all but one respondent in the Airport sample indicated aircraft-related fumes, as opposed to no such response from those in the City sample. For the noise case, only one respondent indicated aircraft-related noise in the city sample, while all but two from the Airport sample failed to make such a reference.

## 5. Conclusions

To conclude, the findings indicate that subjective measures of both exposure to environmental factors (i.e., annoyance) and of health (i.e., HRQOL) are consistent with findings based upon objective measures. This indicates that subjective measurement approaches may constitute promising tools to use when protecting public health. Future studies should include moderating variables such as coping [60] and how the physical environment and sociocultural contexts influences cumulative exposures and human response [61] when investigating the relationships between co-existing pollutant sources and health outcomes.

## Figures and Tables

**Table 1:** Demographic profiles for the six samples. The values are raw frequencies with percentages presented in brackets. Note that percentages may be affected by missing data. Differences in proportions between matched samples within a dataset are tested using Pearson’s chi-square (χ^2^) tests.

Samples	Auckland Samples			Wellington Samples		
	Motorway	Non-Motorway	CBD-Suburb	Airport	Non-Airport	Rural
**Sex**						
Male	93 (34.6)	105 (43)	65 (37.6)	28 (32.6)	31 (33.3)	69 (40.4)
Female	171 (63.6)	140 (57)	107 (61.8)	58 (67.4)	61 (65.6)	98(57.3)
Chi-Square	(χ^2^ (2) = 3.29, *p* = 0.078)			(χ^2^ (1) = 0.05, *p* = 0.824)		
**Age Group (Years)**						
18–20	7 (2.6)	4 (1.6)	3 (1.7)	3 (3.4)	2 (2.2)	2 (1.2)
21–30	36 (13.4)	14 (5.5)	15 (8.7)	7 (8)	8 (8.6)	2 (1.2)
31–40	47 (17.5)	68 (26.9)	22 (12.7)	16 (18.4)	18 (19.4)	22 (12.9)
41–50	55 (20.4)	56 (22.1)	25 (14.5)	16 (18.4)	20 (21.5)	57 (33.3)
51–60	47 (17.5)	40 (15.8)	39 (22.7)	14 (16.1)	20 (21.5)	46 (26.9)
61–70	35 (13)	43 (17.0)	39 (22.7)	16 (18.4)	16 (17.2)	32 (18.7)
70+	37 (13.8)	23 (9.1)	29(16.9)	14 (16.1)	8 (8.6)	10 (5.8)
Chi-Square	(χ^2^ (7) = 18.51, *p* = 0.005)			(χ^2^ (7) = 4.527, *p* = 0.75)		
**Noise Sensitivity**						
Low	98 (38)	91 (34.9)	67 (38.7)	40 (46)	39 (41.9)	66 (38.6)
Moderate	125 (50)	139 (53.3)	81(46.8)	33 (37.9)	41 (44.1)	79 (46.2)
High	26 (10.4)	31 (11.9)	23 (13.3)	14 (16.1)	13 (14)	23 (13.5)
Chi-Square	(χ^2^ (2) = 1.159, *p* = 0.56)			(χ^2^ (2) =7.15, *p* = 0.699)		

**Table 2:** Bivariate and partial correlation coefficients representing the relationship between air pollution annoyance and noise annoyance. Partial correlation controls for Age, Education, Gender, Illness and Noise Sensitivity. All coefficients are statistical significant (*p* < 0.001).

Sample	Auckland Samples			Wellington Samples		
	Motorway	Non-Motorway	CBD-Suburb	Airport	Non-Airport	Rural
*r*	0.566	0.317	0.537	0.630	0.461	0.377
*Partial*	0.532	0.292	0.456	0.633	0.492	0.355

**Table 3:** Results of multiple linear regression analysis (* *p* < 0.05, ** *p* < 0.001).

**PHYSICAL HRQOL**					
**Predictors**	***R***	***R*^2^**	**∆*R*^2^**	***B* (SE)**	***B***
Step 1	0.446	0.199	0.199 **		
Age				0.035 (0.093)	0.012
Education				0.683 (0.160)	0.125 **
Gender				0.385 (0.280)	0.040
Illness				3.937 (0.300)	0.397 **
Noise Sensitivity				−0.693 (0.206)	−0.099 **
Step 2	0.482	0.132	0.033 **		
Air pollution Annoyance				−0.579 (0.124)	−0.131 **
Noise Annoyance				−0.507 (0.121)	−0.117 **
Step 3	0.484	0.134	0.002		
Air × Noise Annoyance				−0.103 (0.087)	−0.096
**PSYCHOLOGICAL HRQOL**					
**Predictors**	***R***	***R*^2^**	**∆*R*^2^**	***B* (SE)**	***B***
Step 1	0.305	0.093	0.093 **		
Age				0.365 (0.077)	0.155 **
Education				0.658 (0.133)	0.154 **
Gender				0.170 (0.232)	0.023
Illness				1.668 (0.249)	0.215 **
Noise Sensitivity				−0.728 (0.170)	−0.133 **
Step 2	0.339	0.115	0.022 **		
Air pollution Annoyance				−0.321 (0.120)	−0.096 *
Noise Annoyance				−0.260 (0.117)	−0.180 *
Step 3	0.341	0.116	0.001		
Air × Noise Annoyance				−0.091 (0.081)	−0.117
**SOCIAL HRQOL**					
**Predictors**	***R***	***R*^2^**	**∆*R*^2^**	***B* (SE)**	***B***
Step 1	0.188	0.035	0.035 **		
Age				0.162 (0.055)	0.096
Education				0.367 (0.095)	0.125 **
Gender				0.338 (0.166)	0.066
Illness				0.609 (0.178)	0.114 **
Noise Sensitivity				−0.176 (0.122)	−0.046
Step 2	0.237	0.056	0.021 **		
Air pollution Annoyance				−0.186 (0.087)	−0.080 *
Noise Annoyance				−0.207 (0.084)	−0.092 *
Step 3	0.239	0.057	0.001		
Air × Noise Annoyance				0.049 (0.057)	0.091
**ENVIRONMENTAL QOL**					
**Predictors**	***R***	***R*^2^**	**∆*R*^2^**	***B* (SE)**	***B***
Step 1	0.297	0.088	0.088 **		
Age				0.606 (0.099)	0.201 **
Education				1.016 (0.171)	0.187 *
Gender				0.699 (0.300)	0.073 *
Illness				1.790 (0.322)	0.180 **
Noise Sensitivity				−0.603 (0.221)	−0.086 *
Step 2	0.410	0.168	0.080 **		
Air pollution Annoyance				−0.509 (0.152)	−0.118 **
Noise Annoyance				−0.891 (0.146)	−0.213 **
Step 3	0.413	0.171	0.002		
Air × Noise Annoyance				−0.167 (0.102)	−0.167
